# Brain Modulatory Effects by Low-Intensity Transcranial Ultrasound Stimulation (TUS): A Systematic Review on Both Animal and Human Studies

**DOI:** 10.3389/fnins.2019.00696

**Published:** 2019-07-24

**Authors:** Pu Wang, Jiaqi Zhang, Jiadan Yu, Colin Smith, Wuwei Feng

**Affiliations:** ^1^Department of Rehabilitation Medicine, Ruijin Hospital, Shanghai Jiaotong University School of Medicine, Shanghai, China; ^2^Department of Rehabilitation Sciences, The Hong Kong Polytechnic University, Hong Kong, China; ^3^School of Rehabilitation Sciences, West China School of Medicine, Sichuan University, Chengdu, China; ^4^Department of Neurology, Medical University of South Carolina, Charleston, SC, United States

**Keywords:** ultrasound, neuromodulation, human, animal, neurostimulation

## Abstract

**Background and objective:** Low Intensity Transcranial Ultrasound Stimulation (TUS) is a new form of non-invasive brain modulation with promising data; however, systematic reviews on the brain modulatory effects of TUS on both animals and humans have not been well-conducted. We aimed to conduct a systematic review on the studies using the TUS to modulate the brain functions and associated behavioral changes in both animals and humans.

**Methods:** A literature search for published studies in the past 10 years was conducted. Two authors independently reviewed the relevant articles. Data were extracted and qualitatively summarized. Quality of studies was assessed by the SYRCLE's risk of bias tool for preclinical studies or the PEDro scale for clinical studies.

**Results:** A total of 24 animal studies (506 animals) and 11 human studies (213 subjects) were included. Findings based on most animal studies demonstrated the excitatory or suppressive modulatory effects of ultrasonic stimulations on motor cortex, somatosensory cortex, thalamus, prefrontal cortex, auditory, and visual areas. Brain modulatory effects also were found among healthy human subjects in seven studies and two clinical studies suggested TUS may result in potential benefits on patients with disorder of consciousness or chronic pain. The safety concerns of TUS seem to be minor based on the human studies.

**Conclusions:** TUS appears to be a viable technique in modulating the brain functions; however, research on TUS is still in its early stages, especially in human studies. Parameters need to be optimized before launching systematic investigations in humans.

## Introduction

Ultrasonic stimulation is a form of mechanical energy like sound, but at frequencies above auditory threshold, i.e., 20,000 HZ to several 100 MHZ, which offers deep penetration (10 to 15 cm or more) with high anatomic specificity (Bystritsky et al., 2011). This technique could be superior to other brain stimulation modalities, such as transcranial magnetic stimulation or transcranial direct current stimulation, in terms of spatial resolution (Bystritsky et al., 2011). Additionally, ultrasonic stimulation can be compatible with simultaneous neuroimaging scanning. Ultrasound can generate various thermal and non-thermal effects at cellular and tissue levels depending on various parameters, including frequency, intensity, pulse repetition frequency, duty cycle, and duration. Based on the frequency, ultrasound can be divided into either focused ultrasound (frequency < 1 MHZ) or unfocused ultrasound (frequency from 1 to 15 MHZ). Focused ultrasound is promising for transcranial neuromodulation because it has less energy aberration. Unfocused ultrasound is typically used for imaging and diagnosis, while recent evidence also finds its neuromodulatory effect on humans (Hameroff et al., 2013; Gibson et al., 2018). Based on the intensity, high intensity (>200 W/cm^2^) ultrasound relies on its thermal properties, which has been used in neurosurgery for tissue ablation. Medium intensity (100–200 W/cm^2^) ultrasound may be used to open the blood-brain barrier (BBB). Low intensity (<100 W/cm^2^) ultrasound mainly depends on its mechanical bioeffects. Although the nature of such effects is not fully understood, experimental studies on animals have suggested that the neuromodulatory effect of TUS is dependent on the mechanosensitive ion channels in the cellular membranes (Kubanek, 2018).

Recently, researchers have been deploying the low intensity transcranial ultrasound stimulation (TUS) to reversibly modulate the neuronal activity without accumulating significant thermal energy over the scalp or in the brain. In animal studies, low intensity TUS have been shown to modulate the brain activities measured by functional magnetic resonance imaging (fMRI) (Yoo et al., 2011a) and cortical oscillations, measured using electroencephalography (EEG) (Kim et al., 2015). Several prior experimental human studies have also showed that low intensity TUS is associated with modulating the excitability of motor cortex, measured using motor evoked potentials (MEP) (Legon et al., 2018b), or sensory evoked neural oscillations, measured using somatosensory evoked potentials (SEP) (Legon et al., 2014). Overall, low intensity TUS could emerge as a promising neuromodulatory tool to induce both brain plasticity and improve mental states and cognition in either healthy individuals or patients with diseases conditions.

To better understand the brain modulatory effects of low-intensity TUS in both animals and humans and to identify gaps and issues in translational research, we aimed to conduct a systematic review on the application of TUS in both animal and human studies with neuroimaging, neurophysiological, neurobehavioral and self-report of mental states data.

## Methods

The Preferred Reporting Items for Systematic Reviews and Meta-analyses (PRISMA) criteria including the “extension” for systematic reviews were followed (Moher et al., 2009). A literature search for studies was conducted on PubMed, Medline, EMBASE and Web of Science using combinations of the following key words: transcranial ultrasound, transcranial focused ultrasound and neuromodulation. Two authors (PW and JZ) independently reviewed the relevant articles. Relevant articles' publication dates were limited to the last 10 years (from July 1st, 2008 to January 18th, 2019) as TUS is a relatively new emerging neural technique.

### Inclusion and Exclusion Criteria

We followed the PICOS framework to organize our inclusion criteria. Studies meeting all of the following inclusion criteria were selected for this review: (1) Population (P): Studies using animals or human subjects as their experimental subjects; (2) Intervention (I): Studies using low-intensity TUS, either focused or unfocused TUS, to modulate the brain functions; (3) Outcomes (O): Studies providing at least one outcome measurement evaluating the neuromodulatory effects of TUS on the brain, including but not limited to neurophysiological measurements (e.g., single-neuron recording, EEG, and TMS outcomes), neuroimaging examinations (e.g., functional magnetic resonance imaging [fMRI] and positron emission tomography [PET]), neurobehavioral changes in sensory, motor, cognition or other domains, and self-report mental state; and (4) Published in the English language. We did not exclude studies based on study design (i.e., with or without sham TUS control).

Studies meeting any of the following criteria were excluded: (1) Study investigating the diagnostic aspect of TUS rather than therapeutical effects; (2) Study investigating the application of TUS in ablation neurosurgery; (3) Study focusing on the effect of ultrasound on neuronal tissues (*in vitro* study); (4) Study evualating the effect of TUS on opening the BBB and enhancing the drug delivery; (5) Study only providing surrogate biomarkers and (6) Study published as the conference abstract without a full text, as dissertation or those published in books.

### Data Extraction

After identifying relevant articles, two authors (PW and JZ) independently extracted the following information from each article: (1) authors and publication year; (2) the type of experimental animals; (3) the protocol of TUS; and (4) the outcomes and main finding of each study. For human studies, we also qualitatively summarized the safety issues regarding the application of TUS.

### Methodological Quality Assessment of Included Studies

Two authors (WP and JY) used the Systematic Review Center for Laboratory Animal Experimentation (SYRCLE) risk of bias tool to assess the studies risk of bias (Hooijmans et al., 2014). The SYRCLE's risk of bias tool analysis 10 items related to selection bias (items 1, 2, 3), performance bias (items 4 and 5), detection bias (items 6 and 7), reporting bias (item 9) and other bias (item 10). These 10 items include item 1: sequence generation, item 2: baseline characteristics, item 3: allocation concealment, item 4: random housing, item 5: blinding, item 6: random outcome assessment, item 7: blinding, item 8: incomplete outcome data, item 9: selective outcome reporting and item 10: other sources of bias. A positive (“yes”) judgement indicates low risk of bias; a negative (“no”) judgement indicates high risk of bias; and an imprecise (“unclear”) judgement were assessed when insufficient details were not reported. Two independent authors (PW and JY) did the assessment, and disagreements were solved through consensus by a third author (JZ).

We assessed the methodological quality of the included human controlled trials using the Physiotherapy Evidence Database (PEDro) scale. The PEDro scale consists of 10 items including random allocation, concealment of allocation, baseline equivalence, blinding procedure, intention to treat analysis, adequate follow-up, between-group statistical analysis, measurement of data variability, and point estimates. Studies with a PEDro score more than 6 were considered good-quality (Bhogal et al., 2005). Scoring discrepancies were resolved with a third author (JZ) if there is any.

## Results

### Characteristics of Studies

**Figure 1:**
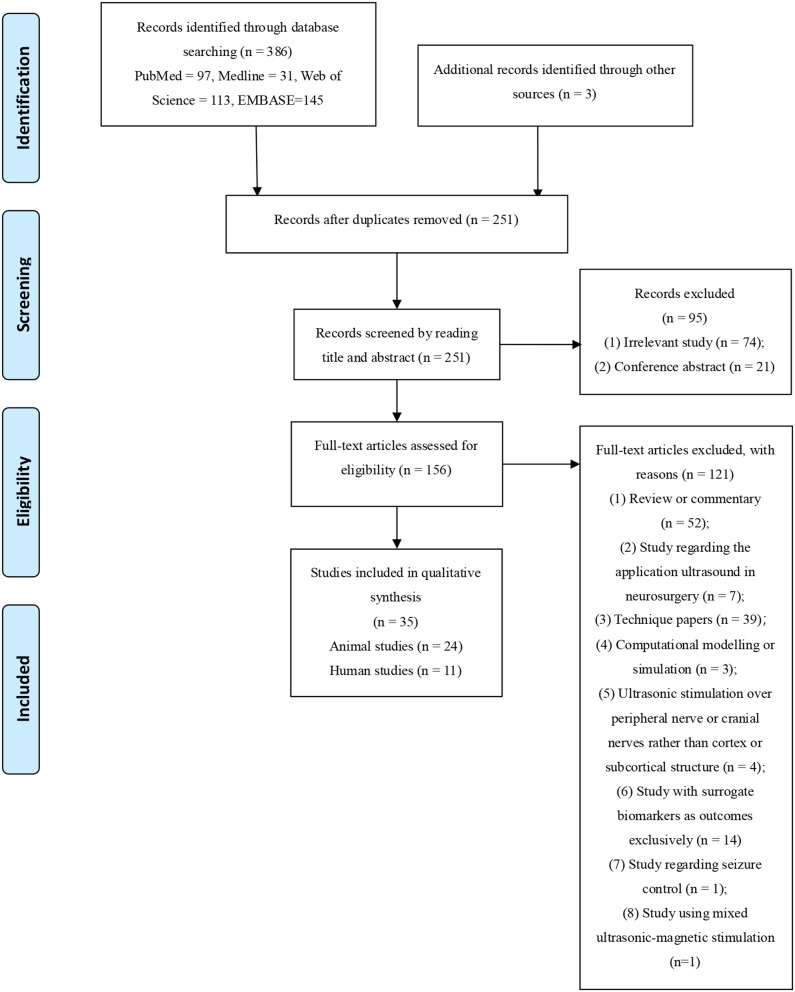
Flowchart of literature search.

Figure 1 showed the identification process for the selection of studies. Briefly, the initial search retrieved 386 manuscripts. After removing duplications, the remaining 256 articles were further screened by reading the title and abstract, of which 95 were excluded because they were irrelevant articles or published as conference abstracts. A total of 156 articles were subjected to full-text review, of which 121 articles were removed for the following reasons: review or commentary (*n* = 52), study regarding the application of ultrasound in neurosurgery (*n* = 7), technique papers regarding the design of ultrasound system, stimulation protocol, parameter optimization and properties improvement (*n* = 39), computational modeling or simulation study (*n* = 4), ultrasonic stimulation targeting peripheral or cranial nerves rather than cortical and subcortical structures (*n* = 4), study with surrogate biomarkers as outcomes exclusively (*n* = 14), study regarding seizure control (*n* = 1) and study using mixed ultrasound magnetic stimulation (*n* = 1). Ultimately 35 studies (Tufail et al., 2010; Yoo et al., 2011a,b, 2018; Deffieux et al., 2013; Hameroff et al., 2013; Kim et al., 2013, 2014a,b, 2015; Legon et al., 2014, 2018a,b; Chu et al., 2015; Guo et al., 2015, 2018; Lee et al., 2015, 2016a,b,c; Ai et al., 2016, 2018; Monti et al., 2016; Yu et al., 2016; Wattiez et al., 2017; Dallapiazza et al., 2018; Daniels et al., 2018; Gibson et al., 2018; Sato et al., 2018; Xie et al., 2018; Yang et al., 2018; Zhang et al., 2018; Li et al., 2019; Sharabi et al., 2019) were selected for inclusion in this review, including 24 animal and 11 human studies.

### Neuromodulation Induced by TUS in Animals

Characteristics of included animal studies are shown in Table 1. Heterogeneities among the included studies can be noted in brain targets, outcomes measurements for modulatory functions, parameters of TUS and characteristics of experimental subjects. Brain targets varied from study to study, including motor cortex (Tufail et al., 2010; Yoo et al., 2011a; Kim et al., 2014a,b; Lee et al., 2014, 2016c), somatosensory cortex (Lee et al., 2014, 2016c; Guo et al., 2018; Xie et al., 2018; Li et al., 2019), thalamus (Yoo et al., 2011b; Dallapiazza et al., 2018), prefrontal (Deffieux et al., 2013; Wattiez et al., 2017), visual areas (Yoo et al., 2011a; Kim et al., 2015; Lee et al., 2016c; Sato et al., 2018), auditory areas (Daniels et al., 2018), and medulla oblongata region (Sharabi et al., 2019). Excitatory effects of TUS in normal animals were found in 14 studies, as represented in limb movements (Tufail et al., 2010; Kim et al., 2014a,b), electromyography (EMG) (Lee et al., 2014; Sato et al., 2018; Sharabi et al., 2019), fMRI (Yoo et al., 2011a; Yang et al., 2018), PET (Kim et al., 2013), EEG power spectrum (Yu et al., 2016), visual evoked potential (VEP) (Kim et al., 2015; Lee et al., 2016c), neuronal recordings (Wattiez et al., 2017; Guo et al., 2018; Li et al., 2019), and recovery time after anesthesia (Yoo et al., 2011b). Inhibitory effects in normal animals were also found in 6 studies, as represented by suppression of SEP, VEP, and auditory evoked potential (AEP) (Yoo et al., 2011a, 2018; Chu et al., 2015; Kim et al., 2015; Dallapiazza et al., 2018; Daniels et al., 2018). One study showed that TUS altered the cortico-muscular coupling (i.e., coupling relationship between stimulated motor cortex and effector muscle), which was significantly enhanced with the increase of the number of tone bursts applied (Xie et al., 2018). Experimental rats with diseases conditions were used in three articles: the first reported that 360 trials (400 ms per trial) ultrasonic stimulation over the ischemic core at an intensity of = 2.155 W/cm^2^ (spatial peak pulse average intensity, ISPPA) significantly reduced the volume of lesion and improved the neurological outcomes in stroke rats, as comparison to the sham stimulation (Guo et al., 2015), the second showed that prefrontal 2-week ultrasonic stimulation at an intensity of 7.59 W/cm^2^ (ISPPA) improved the depression-like behaviors of depressed rats (Zhang et al., 2018) and the third reported that TUS at an intensity of 27.2 W/cm^2^ (ISPPA) over the medulla oblongata region could suppress the essential tremors (Sharabi et al., 2019) in Harmaline-induced rats.

**Table 1:** Characteristics of included animal studies.

**References**	**Experimental subjects**	**Protocol of TUS**	**Brain targets**	**Outcomes of interest**	**Major findings**
Tufail et al. (2010)	Normal mice (*n =* 11)	Frequency: 0.25 to 0.5 MHZ; Intensity: 0.075 to 0.229 W/cm^2^ (ISPPA); Pulse repetition frequency: 1.2 to 3 kHZ; Duty cycle: 19 to 86%; Sonication duration: 26 to 333 ms;	Motor cortex	Behavior: EMG, Rotorod task and wire-hanging task;	(1) Ultrasonic stimulation of the motor cortex evoked motor behaviors; (2) No significant effects on Rotorod task and wire-hanging task.
Yoo et al. (2011a)	Normal rabbits (*n =* 19)	Motor paradigm: Frequency: 0.69 MHz; Intensity: 3.3, 6.4, 9.5, and 12.6 W/cm^2^ (ISPPA); Pulse repetition frequency: 0.01 kHZ; Duty cycle: 50%; Sonication duration: 500, 1,000, 1,500, and 2,000 ms; Suppression paradigm: Frequency: 0.69 MHz; Intensity: 3.3 and 6.4 W/cm^2^ (ISPPA); Pulse repetition frequency: 0.1 kHZ; Duty cycle: 5%; Sonication duration: > 7,000 to 8,000 ms;	Motor cortex and visual areas	Neuroimaging: fMRI;	TUS had bimodal modulatory effects: (1) Motor paradigm: ultrasound induced the motor cortex activation and detectable motor activity (ISPPA = 12.6 W/cm^2^); (2) Suppression paradigm: ultrasound reduced the magnitude of P30 VEP component (ISPPA = 3.3 W/cm^2^).
Yoo et al. (2011b)	Normal rats (*n =* 17)	Frequency: 0.65 MHZ Intensity: 3.3 W/cm^2^ or 6 W/cm^2^ (ISPPA); Pulse repetition frequency: 0.1 kHZ; Tone burst duration: 0.5 ms;	Thalamus	Behavior: Time to voluntary movement from anesthesia; anesthetic duration;	(1) Ultrasonic stimulation significantly reduced the time to show pinch response and voluntary movement; (2) A higher intensity of 6 W/cm^2^ (ISPPA) significantly decreased anesthetic duration.
Deffieux et al. (2013)	Normal macaque monkey (*n* = 2)	Frequency: 0.32 MHZ; Intensity: 4 ± 1.1 W/cm^2^ (ISPPA); Duty cycle: 100%; Sonication duration: 100 ms;	Left FEF and Premotor cortex;	Behavior: Antisaccade task;	Ultrasonic stimulation significantly modulated antisaccade task latencies.
Kim et al. (2013)	Normal rats (*n =* 17)	Frequency: 0.35 MHZ Intensity: 3 W/cm^2^ (ISPTA); Pulse repetition frequency: 1 kHZ; Tone burst duration: 0.5 ms; Sonication duration: 300 ms;	Unilateral hemisphere	Neuroimaging: PET (F-FDG uptake);	Spatially distinct increases of the glucose metabolic activity was present only at the center of stimulation focus.
Kim et al. (2014a)	Normal rats (*n =* 7)	Frequency: 0.35 MHZ Intensity: 3 W/cm^2^ (ISPTA); Pulse repetition frequency: 1 kHZ; Duty cycle: 50%; Sonication duration: 300 ms;	Motor cortex	Behavior: Tail movement; Neuroimaging: PET (F-FDG uptake);	(1) The size of the neuromodulatory area was found to be much smaller than the size of the acoustic focus; (2) The average delay in motor response was measured to be 171 ± 63 ms from the onset of sonication.
Kim et al. (2014b)	Normal rats (*n =* 37)	Frequency: 0.35 and 0.65 MHZ Intensity: 4.9-22.4 W/cm^2^ (ISPTA); Pulse repetition frequency: 0.06 to 2.8 kHZ; Duty cycle: 30 to 100% Sonication duration: 150-400 ms;	Motor cortex	Behavior: Tail movement;	Movement was elicited at minimum threshold intensities of 4.9–5.6 W/cm^2^ (ISPPA) in 50% of duty cycle, and 300 ms of sonication duration, at 0.35 kHz.
Kim et al. (2015)	Normal rats (*n =* 24)	Frequency: 0.35 MHZ Intensity: 1, 3, and 5 W/cm^2^ (ISPPA); Pulse repetition frequency: 0.1 kHZ; Duty cycle: 1, 3, 5, and 8.3% Tone-burst duration: 0.5 ms; Sonication exposure: 150 s;	Visual area	Neuro-oscillation: EEG (VEP);	(1) The magnitude of VEP was suppressed during the sonication using a 5% duty cycle and an intensity of 3 W/cm^2^ (ISPPA); however, this suppressive effect was not present when using a lower intensity and duty cycle; (2) A higher intensity and duty cycle resulted in a slight elevation in VEP magnitude.
Chu et al. (2015)	Normal rats (*n =* 118)	Frequency: 0.4 MHZ; Intensity: 0.3, 0.55 and 0.8 (MI); Pulse repetition frequency: 0.01 kHZ; Duty cycle: 1%; Sonication duration: 10 ms; Sonication exposure: 120 s;	left primary somatosensory cortex	Neuro-oscillation: EEG (SSEP); Neuroimaging: fMRI (BOLD);	(1) 0.8-MI TUS profoundly suppressed SSEP amplitude and prolonged latency for 7 days; 0.55-MI TUS resulted in short-term suppression of SSEP for < 60 min and did not affect latency. No significant change was observed for the 0.3-MI and control groups. (2) BOLD responses were reduced for 2 days for the 0.8-MI group; transiently reduced for the 0.55-MI group and was not observed for the 0.3- MI and control groups.
Guo et al. (2015)	Ischemic stroke rats (*n =* 38)	Frequency: 0.5 MHZ; Intensity: 0.57 and 0.86 W/cm^2^ (ISPTA); Pulse repetition frequency: 1.5 kHZ; Number of acoustic cycles per pulse: 200; Sonication duration: 400 ms per trial; Sonication exposure: 144 s (totally 360 trials);	Ischemic core	Structure: Lesion volume; Behavior: NSS;	(1) Ischemic lesion was significantly reduced after receiving TUS; (2) The cortical infarct volume of animals in the control group was more than 3 fold of that in the TUS group; (3) Animals in the TUS group showed significantly lower NSS than that in the control group.
Lee et al. (2014)	Normal sheep (*n =* 8)	Frequency: 0.25 MHZ; Intensity: 1.4 to 15.5 W/cm^2^ (ISPPA); Pulse repetition frequency: 0.5 kHZ; Duty cycle: 50%; Sonication duration: 50 to 150 ms;	Sensorimotor cortex	Neuropsychological index: MEPs;	(1) A MEP from the hind leg muscle contralateral to the sonicated hemisphere was detected when using an intensity of 6.9 W/cm^2^ (ISPPA).
Lee et al. (2016c)	Normal sheep (*n =* 8)	Frequency: 0.25 MHZ; Intensity: 1.7 to 14.3 W/cm^2^ (ISPPA); Pulse repetition frequency: 0.5 kHZ; Duty cycle: 50%; Sonication duration: 300 ms;	Primary sensorimotor and visual areas	Neuropsychological index: MEPs; Neuro-oscillation: EEG (VEP);	(1) Sonication over the primary sensorimotor areas elicited electromyographic responses from the contralateral hind leg at different intensity thresholds in different sheep; (2) Sonication over the visual areas generated VEPs at different intensity thresholds in different sheep.
Yu et al. (2016)	Normal rats (*n =* 3)	Frequency: 0.5 MHZ; Intensity: 0.01 W/cm^2^ (ISPTA); Pulse repetition frequency: 2 kHZ; Sonication duration: 5 and 200 ms;	Multiple-site (16 scalp EEG electrodes)	Neuro-oscillation: EEG (ESI);	TUS activated the stimulation site and the activation propagating to surrounding areas over time, denoted by ESI.
Wattiez et al. (2017)	Normal macaque monkey (*n =* 2)	Frequency: 0.32 MHZ; Intensity: 1.9 and 5.6 W/cm^2^ (ISPPA); Duty cycle: 100%; Sonication duration: 100 ms;	FEF	Neuronal activity: Single-neuron recording (during an antisaccade task);	Supplementary eye field activity was significantly increased shortly after TUS.
Dallapiazza et al. (2018)	Normal Yorkshire swine (*n =* 10)	**Frequency**: 1.14 MHZ; Intensity: 25–30 W/cm^2^ (ISPPA); Pulse repetition frequency: 0.01 kHZ; Duty cycle: 43.7%; Sonication duration: 43.7 ms; Sonication exposure: 40 s;	Sensory thalamus; ventroposterolateral thalamic nucleus	Neuro-oscillation: EEG (SSEP);	Ultrasonic stimulation suppressed the SSEP (trigeminal-evoked or tibial-evoked).
Guo et al. (2018)	Normal guinea pigs (*n =* 2)	Frequency: 0.22 MHZ; Intensity: 0.02 W/cm^2^ (ISPPA); Pulse repetition frequency: 1 kHZ; Sonication duration: 500 ms;	Primary somatosensory cortex, primary auditory cortex and visual cortex;	Neuronal activity: Neural recording;	(1) Ultrasonic stimulation elicited extensive activation across cortical and subcortical brain regions. (2) Transection of the auditory nerves or removal of cochlear fluids eliminated the US-induced activation.
Sato et al. (2018)	Transgenic Thy1-GCaMP6s mice (intact and deafened) (*n =* 20)	Frequency: 0.5 MHZ; Intensity: 0.034 to 4.2 W/cm^2^ (ISPTA); Pulse repetition frequency: 1.5 kHZ; Sonication duration: 80 ms;	Primary somatosensory cortex, primary auditory cortex and visual cortex;	Neuronal activity: Neural recording; Behavior: EMG;	Both ultrasound and audible sound elicited motor responses, with both responses reduced by chemical deafening.
Yang et al. (2018)	Normal macaque monkey (*n =* 2)	Frequency: 0.25 MHZ; Intensity: 29.5 W/cm^2^ (ISPPA); Pulse repetition frequency: 2 kHZ; Duty cycle: 50%; Sonication duration: 300 ms Sonication exposure: 3 s (10 sonications);	Primary somatosensory cortex	Neuroimaging: fMRI;	(1) Tactile stimulation-and TUS evoked similar fMRI activation patterns; (2) FUS conditions also indicated that TUS modulated the tactile network differently;
Yoo et al. (2018)	Normal rats (*n =* 11)	Frequency: 0.65 MHZ; Intensity: 4.2 W/cm^2^ (ISPPA); Pulse repetition frequency: 0.1 kHZ; Duty cycle: 5% Sonication exposure: 30-min;	Somatosensory areas	Neuro-oscillation: EEG (SEP);	SEP changes were found beyond 35-min after TUS;
Zhang et al. (2018)	Depressed rats (*n =* 76)	Frequency: 0.5 MHZ; Intensity: 7.59 W/cm^2^ (ISPPA); Pulse repetition frequency: 1.5 kHZ; Duty cycle: 60%; Sonication duration: 400 ms; Sonication exposure: 15-min per day for 2 weeks;	Prefrontal cortex	Behavior: Sucrose Preference Test, Open-field Test and Forced Swimming Test;	Recovery of depression-like phenotypes, i.e., anhedonia and reduced exploratory behaviors was found after TUS
Li et al. (2019)	Normal mice (*n =* 17)	Frequency: 2 MHZ; Intensity: 46 W/cm^2^ (ISPPA); 0.70 W/cm^2^ (ISPTA) Pulse repetition frequency: 1 kHZ; Duty cycle: 30%; Sonication duration: 300 ms; Sonication exposure: 360 s;	Primary somatosensory cortex	Neuronal activity: Neural recording; Behavior: Head-turning behaviors;	TUS induced action potentials and evoked head-turning behaviors.
Xie et al. (2018)	Normal mice (*n =* 9)	Frequency: 0.5 MHZ; Intensity: 1.10 W/cm^2^ (AI); Pulse repetition frequency: 1 kHZ; Number of acoustic cycles per pulse: 250; Number of tone bursts: 100, 150, 200, 250 and 300;	Primary motor cortex	Neuronal activity: Local filed potential; Cortico-muscular coupling assessed by mutual information and transfer entropy; Behavior: EMG;	TUS altered the cortico-muscular coupling which was significantly enhanced with the increase of NTB.
Daniels et al. (2018)	Normal rats (*n =* 22) and pig (*n =* 5)	Frequency: 0.23 MHZ; Intensity: 2.3 W/cm^2^ and 4.6 W/cm^2^ (ISPPA) Pulse repetition frequency: 1 kHZ; Duty cycle: 3%; Sonication duration: 100 ms; Sonication exposure: 52 s;	Inferior colliculus (rats) Auditory cortex region (pigs)	Neuro-oscillation: AEPs;	(1) TUS suppressed the AEPs in all animals; (2) The suppressive effect was weaker for rats treated at 2.3 W/cm^2^ than that treated at 4.6 W/cm^2^.
Sharabi et al. (2019)	Harmaline-induced rats TUS, *n =* 5 and sham, *n =* 8) and normal rats (TUS, *n =* 5, and sham, *n =* 3)	Frequency: 0.23 MHZ; Intensity: 27.2 W/cm^2^ (ISPPA); Pulse repetition frequency: 1 kHZ; Duty cycle: 100%; Sonication duration: 100 ms; Sonication exposure: 52 s;	Medulla oblongata region	Behavior: EMG;	(1) TUS induced tremor suppression in 12 out of 13 Harmaline-induced rats; (2) TUS induced motor response which was synchronized with the sonication in both Harmaline-induced rats and normal rats.

*ISPPA, Intensity spatial peak pulse average; EMG, Electromyography; fMRI, Functional magnetic resonance imaging; ISPTA, Intensity spatial peak time average; FEF, Frontal eye field; PET, Positron emission tomography; FDG, 18-fludeoxyglucose; VEP, Visual evoked potential; SSEPs, Somatosensory evoked potentials; BOLD, Blood-oxygen-level dependent; NSS, Neurological severity score; MEP, Motor evoked potential; ESI, Electrophysiological source imaging; SEP, Somatosensory evoked potential; AI, Auditory intensity; AEP, Auditory evoked potential; NTB, Number of tone burst*.

Two studies used the non-human primates (i.e., macaque monkey) as the experimental subjects in which TUS at intensities of 1.9 and 5.6 W/cm^2^ (ISPPA) was applied over the frontal eye field. Studies showed that low intensity TUS could inference the performance in an antisaccade task. The real-time neuronal recording showed the accompanying neuronal activation along with sonication (Deffieux et al., 2013; Wattiez et al., 2017). Two studies explored the role of auditory pathway in TUS induced brain modulation and they found the neuronal activation or motor responses induced by sonication could be suppressed by deafening in Guinea pigs or mouse (Guo et al., 2018; Sato et al., 2018).

### Neuromodulation Induced by TUS in Humans

Characteristics of included human studies are shown in Table 2. Heterogeneities among the included studies can be noted in characteristics of human subjects, outcome measurements for modulatory effect, brain targets and parameters of TUS. Nine articles recruited healthy subjects (Legon et al., 2014, 2018a,b; Lee et al., 2015, 2016a,b; Ai et al., 2016, 2018; Gibson et al., 2018) and two studies focused on subjects with traumatic head injury and chronic pain (Hameroff et al., 2013; Monti et al., 2016). The excitatory effects of TUS also were identified in human subjects, as represented in fMRI (Ai et al., 2016, 2018; Lee et al., 2016b), motor evoked potential (MEP) (Gibson et al., 2018) and EEG (Legon et al., 2018b). Some studies showed the effects of TUS on reducing the SEP (Legon et al., 2014, 2018a), MEP (Ai et al., 2018; Gibson et al., 2018; Legon et al., 2018b), and intracortical facilitation (ICF) (Legon et al., 2018b). The modulated brain targets included motor cortex (Ai et al., 2016, 2018; Gibson et al., 2018; Legon et al., 2018b), somatosensory (Lee et al., 2014, 2015, 2016a), thalamus (Monti et al., 2016; Legon et al., 2018a), caudate nuclei (Ai et al., 2016), and visual cortex (Lee et al., 2016b). Four articles showed the effects of TUS on subjective perception and behavioral performance, such as inducing body sensations (Lee et al., 2015, 2016a), inferencing the two-point discrimination tasks (Legon et al., 2014) and altering the reaction time (Legon et al., 2018b). Hameroff et al. reported the effect of low-intensity ultrasonic stimulation (frequency = 8 MHz; intensity = 0.152 W/cm^2^ [Spatial-peak temporal-average intensity, ISPTA] and 15 s sonication exposure) over the posterior frontal cortex on patients with chronic pain (Hameroff et al., 2013). Results showed that ultrasonic stimulation improved mood and slightly reduced the pain, as comparison to the sham stimulation. A single case study reported by Monti et al. showed that low-intensity ultrasound over the thalamus (frequency = 0.65 MHz; intensity = 0.72 W/cm^2^ [ISPTA] and 10 runs of 30 s sonication) aided in speeding up the recovery of this patient with disorder of consciousness after brain injuries (Monti et al., 2016).

**Table 2:** Characteristics of included human studies.

**References**	**Experimental subjects**	**Protocol of TUS**	**Stimulated brain regions**	**Outcomes of interest**	**Side effects**	**Major findings**
Hameroff et al. (2013)	Patients with chronic pain (*n =* 14)	Frequency: 8 MHz Intensity: 0.7 (MI), 0.152 W/ cm^2^ (ISPTA); Sonication exposure: 15 s;	Right posterior frontal cortex, contralateral to maximal pain side	Clinical scales: Pain (NRS) and mood (VAMS/Global affect);	NR	(1) Mood was improved 10-min and 40-min following TUS compared with placebo. (2) Pain was slightly reduced following TUS at 40-min.
Legon et al. (2014)	Healthy humans (*n =* 10)	Frequency: 0.5 MHz; Intensity: 1.13 (MI); 5.9 W/cm^2^ (ISPPA); Pulse repetition frequency: 1 kHZ; Duty cycle: 36%; Sonication duration: 500 ms;	Primary somatosensory cortex	Neuro-oscillation: EEG (ERP and LP components of SEPs) Behavior: Two-point discrimination tasks;	NR	(1) TUS modulated the amplitudes of both short-latency and late-onset SEP complexes; (2) TUS significantly modulated the power of late-onset alpha-, beta- and gamma-band activity occurring about 200 ms after MN stimulation. (3) TUS enhanced performance on sensory discrimination tasks but did not affect task attention or response bias.
Lee et al. (2015)	Healthy humans (*n =* 18)	Frequency: 0.25 MHz; Intensity: 3W/cm^2^ (ISPPA); 0.7 W/cm^2^ (ISPTA); Pulse repetition frequency: 0.5 kHZ; Duty cycle: 50%; Sonication duration: 300 ms;	Primary somatosensory cortex	Neuro-oscillation: EEG (TUS-induced cortical potentials); Behavior: tactile sensations over the contralateral upper limb;	No side effects	(1) TUS did not elicite explicit tactile sensations; (2) TUS elicited the cortical evoked potentials similar to the SEP generated by MN stimulation.
Lee et al. (2016a)	Healthy humans (*n =* 10)	Frequency: 0.21 MHz Intensity: 3.5 to 4.4 W/cm^2^ (ISPTA); 7.0 to 8.8 W/cm^2^ (ISPPA) Pulse repetition frequency: 0.5 kHZ Duty cycle: 50% Sonication duration: 500 ms;	Primary and secondary somatosensory cortex	Neuroimaging: fMRI; Neuro-oscillation: EEG (TUS-induced cortical potentials); Behavioral changes: tactile sensations;	No side effects	TUS elicited tactile sensations
Lee et al. (2016b)	Healthy humans (*n =* 19)	Frequency: 0.27 MHz Intensity: 0.7 to 6.6 W/cm^2^ (ISPPA); Pulse repetition frequency: 0.5 kHZ Duty cycle: 50% Sonication duration: 300 ms	Visual cortex	Neuro-oscillation: EEG; Neuroimaging: fMRI; Behavior: Phosphene perception;	One subject reported a transient headache during sham TUS	TUS activated the sonicated brain area and elicits the associated efferent sensory perception in the form of phosphene.
Ai et al. (2016)	Healthy humans (*n =* 6)	3T MRI experiment: Frequency: 0.5 MHz; Intensity: 6 W/ cm^2^ (ISPPA); Pulse repetition frequency: 1 kHZ Duty cycle: 36%; Sonication duration: 500 ms; 7T MRI experiment: Frequency: 0.86 MHz; Intensity: 6 W/ cm^2^ (ISPPA); Pulse repetition frequency: 0.5 kHZ; Duty cycle: 50%; Sonication duration: 500 ms;	Primary sensorimotor cortex; Caudate area	Neuroimaging: fMRI (cortical BOLD at 3T and sub-cortical BOLD at 7T);	NR	(1) BOLD activation was detected the primary sensorimotor cortex in the 3T studies; (2) BOLD activation was detected in the caudate in the 7T study.
Monti et al. (2016)	Patients with post TBI disorder of consciousness (*n =* 1)	Frequency: 0.65 MHz; Intensity: 0.72 W/cm^2^ (ISPTA); Pulse repetition frequency: 100 HZ; Duty cycle: 5%; Sonication exposure: 30 s per sonication with totally 10 sonication;	Thalamus	Clinical scales: CRS-R	NR	(1) At 3 days post-ultrasound, the patient demonstrated full language comprehension, reliable response to command, and reliable communication, consistent with emergence from MCS. (2) At 5 days post-TUS, the patient attempted to walk.
Legon et al. (2018a)	Healthy humans (*n =* 50)	Frequency: 0.5 MHz Intensity: 0.9 (MI); 17.12 W/cm^2^; 6.16 W/cm^2^ (ISPTA); Pulse repetition frequency: 1 kHZ; Duty cycle: 36%; Sonication Duration: 500 ms;	Primary motor cortex	Neuropsychological index: recruitment curves; MEPs; SICI; ICF; Behavior: stimulus response reaction time task	Mild and moderate symptoms in some participants	(1) TUS inhibited the amplitude of MEP and attenuates ICF but does not affect SICI; (2) TUS reduced reaction time on a simple stimulus response task.
Legon et al. (2018b)	Healthy humans (*n =* 40)	Frequency: 0.5 MHz; Intensity: 0.89 (MI); 7.02 W/cm^2^ (ISPPA); Pulse repetition frequency: 1 kHZ; Duty cycle: 36%; Sonication Duration: 500 ms; Sonication exposure: 300 ultrasonic waveforms were delivered every 4 s;	Thalamus	Neuro-oscillation: EEG (SEPs and power spectrum) Behavior: Two-point discrimination task	NR	(1) TUS inhibited the amplitude of the P14 SEP as compared to sham. These results were accompanied by alpha and beta power attenuation as well as time-locked gamma power inhibition. (2) Participants performed significantly worse than chance on a discrimination task during TUS.
Ai et al. (2018)	Healthy humans (*n =* 5)	Frequency: 0.5 MHz; Intensity: 16.95 W/cm^2^ (ISPPA); 0.97 (MI) Pulse repetition frequency: 1 kHZ; Duty cycle: 36%; Sonication Duration: 500 ms;	Primary motor cortex	Neuroimaging outcome: fMRI (BOLD during finger tapping)	NR	TUS increased the activation of the targeted finger presentation of M1 but did not extend to functionally connected motor regions.
Gibson et al. (2018)	Healthy humans (Verum: *n =* 19; Sham *n =* 21)	Frequency: 2.32 MHz; Intensity: 34.96 W/cm^2^ (ISPPA); 132.85 W/cm^2^ (ISPTA) Duty cycle: < 1%; Sonication exposure: 2-min;	Primary motor cortex	Neuropsychological index: MEPs;	No side effects	TUS increased the cortical excitability of M1 immediately after stimulation and 6-min later, but not 11-min later.

*MI, Mechanical index; ISPTA, Intensity spatial peak time average; NRS Numerical rating scale for pain, VAMS, Visual analog mood scale; NR, Not reported; TUS, Transcranial focused ultrasound; ISPPA, Intensity spatial peak pulse average; S1, Primary somatosensory cortex EEG, Electroencephalography; ERP, Event-related potential; LP, Late potential; S2, second somatosensory cortex; fMRI, Functional magnetic resonance imaging; BOLD, Blood oxygen level dependent; TBI, Traumatic brain injury; CRS-R, Coma recovery scale-revised; MCS, minimally conscious state; MEP, Motor evoked potential; SICI, Short-interval intracortical inhibition; ICF, Intracortical facilitation; SEP, somatosensory evoked potential; MN, median nerve*.

### Safety Profiles in Humans

Most studies with human subjects reported no side effects caused by TUS, except that Lee et al. reported a case suffered transient headache after the sham TUS session (Lee et al., 2016b) and Legon et al. reported several subjects reported mild to moderate level of symptoms, including neck pain, sleepiness, muscle twitches, itchiness and headache, but all of them were transient (Legon et al., 2018b). In Legon et al. participants received TUS at frequency of 0.5 MHz and at intensities of 17.12 W/cm^2^ (ISPPA) and 6.16 W/cm^2^ (ISPTA) and TMS assessments and the author did not attribute the side effects to TUS. Overall, the percentage of people who suffered side effects was unknown and the causation was also unclear.

### Methodological Quality of Included Studies

Methodological quality of animal studies was assessed by the SYRCLE, with a prevalence of items classified as “unclear” (55.8%) or “no” (43.8%). The average PEDro score for eight of night human trials is 6.9, ranging from 2 to 8. The summarized of methodological quality assessments of animal studies and human studies were provided in Tables S1, S2, respectively.

## Discussion

This systematic review retrieved the published studies in the past 10 years in both animal and humans regarding the investigation of TUS in modulating the brain functions as well as behavioral outcomes.

### Findings on Animals

Several parameters were used in the animal studies, such as, the frequency ranges from 0.25 to 1.14 MHz, and the stimulation duration varies from a single-session to 2-week daily stimulation. Based on current research, we are not sure about the optimal parameters of TUS on neuromodulation, and further investigations are needed. Among the included animal studies, Yoo et al. (2011a) examined both excitatory and inhibitory modulation of TUS in rabbits, they showed that a longer sonication duration was associated with suppressive effects while a shorter sonication duration was associated with excitatory effects, at frequency of 0.69 MHz and at intensities of 3.3 and 6.4 W/cm^2^ (ISPPA). A higher intensity and duty cycle seem to induce the excitatory effects, as revealed by some of our included studies (Yoo et al., 2011a; Kim et al., 2015). However, different combinations of parameters may result in differential modulatory effects. Inter-subject variation in response to TUS also should be noted which has been suggested by some of our included studies (Ai et al., 2016; Lee et al., 2016c). Although the modulatory effects of TUS on the brain has not been fully interpreted, it is still promising in selectively modulating the brain activities with either excitatory or inhibitory effects.

There were two non-human primate studies, in which the neuromodulatory effects of TUS on the prefrontal areas were investigated (Deffieux et al., 2013; Wattiez et al., 2017). Their results showed that ultrasound significantly modulated attention allocation in macaque monkeys, as an example of the cognitive functions (Deffieux et al., 2013). Concurrent neuronal activation during sonication when performing the cognitive tasks, denoted by single-neuron recordings, was found (Wattiez et al., 2017), implicating the future use of TUS in behavioral neuroscience research of large animals.

The quality of animal studies deserves discussions. All of animal studies described the baseline characteristics and completeness of outcome data for each main outcome. About 55% studies report random outcome assessment, but none of studies describe the method used to conceal the allocation sequence, the methods used to generate the sample allocation sequence and clearly. The randomization may be recognized by the investigators, resulting in selective bias. Besides, only 12.5% of studies blind to assessors and 4.1% blind to caregivers and researchers about the intervention. Thus, we need interpret the results with cautions.

### Finding in Humans

Compared with animal studies (*n* = 24), there were much less research on humans (*n* = 11). Only two studies have investigated the effect of TUS on human with disease conditions. Various ultrasound parameters were employed in human studies. For example, in majority of human studies, the stimulating frequency usually was between 0.21 and 0.86 MHZ (Legon et al., 2014, 2018a,b; Lee et al., 2015, 2016a,b; Ai et al., 2016, 2018; Monti et al., 2016), except that there were two studies employed TUS for brain modulation, with a relatively higher frequency (Hameroff et al., 2013; Gibson et al., 2018) (2.32 MHZ by Gibson et al. and 8 MHZ by Hameroff et al.). The heterogeneity of optimal intensity, duty cycle and duration of TUS should be acknowledged when interpreting and comparing results across studies.

It is believed that ultrasound could induce brain activation or suppression via different mechanisms, including activating the voltage-gated sodium and calcium channels and mechano-sensitive ion channels. Specially, ultrasound primarily act via microtubule, which forms part of the cytoskeleton. It was found that the resonant frequencies of microtubules are in a wide range (from 12 kHZ to 30 MHZ) (Hameroff et al., 2013). Hence, the frequencies of both unfocused TUS (1 to 15 MHZ) and focused TUS (<1 MHZ) could be suitable for neuromodulation. Both focused ultrasound (ISPPA = 16.95 W/cm^2^) (Ai et al., 2018) and diagnostic ultrasound (ISPPA = 34.96 W/cm^2^) (Gibson et al., 2018) yielded an after-effect on enhancing the cortical motor excitability in human subjects, indicating the frequency alone may not be a significant parameter to change the properties of brain modulatory effect associating with TUS. However, it should be a concern that TUS at frequency (1 to 15 MHZ) may suffer from greater energy aberration than that at low frequency (<1 MHZ) (Gibson et al., 2018). A systematic comparison of the brain modulatory effects between these two types of TUS is still lacking.

Due to deep penetrating property, thalamus has been a target for ultrasound. Previous animal studies suggested that thalamic ultrasonic stimulation was able to increase the extracellular level of dopamine and serotonin and decrease the extracellular level of γ-aminobutyric acid (Yang et al., 2012). The modification of those major excitatory and inhibitory neurotransmitters is thought to be associated with the modulatory effect of TUS on the activities of neuronal system. A case study by Monti et al. (2016) showed the ultrasound may speed up the recovery of post-trauma unconscious patients; however, there is lack of a control group. The modulatory effect of thalamus ultrasonic stimulation also was revealed by other two studies in animals (Yoo et al., 2011b; Dallapiazza et al., 2018) and one study in humans (Legon et al., 2018a). Those results, therefore, pointed out another potential clinical utility of thalamus ultrasonic stimulation on coma recovery. Among the included human studies, majority of them focused on the motor and somatosensory responses and their corresponding neurophysiological oscillations or neuroimaging change. No study has explored the utility of TUS on inferencing with the performance in cognitive tasks regarding the role of TUS on modulating the high-order cognitive processes in human subjects.

Safety profiles associated with TUS appear to be reasonable. Among the eleven human studies, nine articles did not report the side effects related to TUS. Two articles reported mild and self-limited symptoms only, but we cannot know whether the events were caused by sonication, or just caused by other experimental procedures (e.g., fMRI and TMS). However, we have to interpret the data cautiously as in most studies, the safety data has not collected systematically. Moreover, the potential side effects associating with TUS is all rated by self-report questionnaires in our included studies. Mild changes of mental state or brain structure following TUS might be neglected in this case. Besides, there has not been one phase I dosing and safety study. Serious adverse events might only be detected with large sample size.

Overall, quality of included human studies was good with average PEDro score of 7 (out of 10). However, concealed allocation was not performed in any human studies and the investigators who operated the TUS was only blinded in one study. More robust studies are needed to replicate these research findings.

Overall, several issues need to be further investigated before future human studies can be better carried out: (1) short-term and long-term safety profiles of low intensity TUS on humans, both healthy controls and subjects with disease conditions; (2) optimizing parameters of TUS: delineating the optimal intensity, duty cycle, pulse repetition frequency and duration for various disease conditions; patient selection and dose-response issues; and (3) mechanisms of brain modulatory effects need to be explored at the human levels as well.

Our study is not free from limitations. On one hand, we only included published manuscripts in the English language, and it is possible that we may omit manuscripts published in other languages which may diminish the comprehensiveness of this review. On the other hand, the inability of conducting meta-analysis limited our ability to quantitatively compare results across pre-clinical and clinical studies.

## Conclusion

By conducting this systematic review of both pre-clinical and clinical studies on TUS, we have identified several issues and gaps in translational research in TUS. Overall, low-intensity TUS is a promising non-invasive brain stimulation tool which appears to have neuromodulatory effects on brain functions and associated behavioral changes. Studies in humans either in healthy or diseased conditions are still in an infant stage. Device-related parameter probably needs optimization before launching systematic investigation of TUS applications in humans.

## Data Availability

Data sharing not applicable to this study as no datasets were generated or analysed during the current study.

## Author Contributions

PW and WF: conceptualization. PW, JZ, JY, and WF: methodology and formal analysis. PW, JZ, and JY: investigation and original draft preparation. CS and WF: review and editing. WF: supervision and funding acquisition.

### Conflict of Interest Statement

The authors declare that the research was conducted in the absence of any commercial or financial relationships that could be construed as a potential conflict of interest.
