# Supplementary material for: Brain Modulatory Effects by Low-Intensity Transcranial Ultrasound Stimulation (TUS): A Systematic Review on Both Animal and Human Studies
Source: Front Neurosci. 2019 Jul 24;13:696. doi: 10.3389/fnins.2019.00696 (PMC6667677; doi:10.3389/fnins.2019.00696)
Supplement: Supplementary file 1 [file Table_1.DOCX]

**Supplementary materials**

**Brain Modulatory Effects by Low-Intensity Transcranial Ultrasound Stimulation (TUS): A Systematic Review on both Animal and Human Studies**

Pu Wang^1^ MD PhD, Jiaqi Zhang^2^ MSc, Jiadan Yu^3^ MPhil, Colin Smith^4^ BS, Wuwei Feng^4^* MD MS

| Table S1. Quality Assessment of Included Animals Studies by SYRCLE’s tool | Page 2-3 |
| --- | --- |
| Table S2. Quality Assessment of Included Human Studies by PEDro | Page 4 |

**Table S1. Quality Assessment of Included Animals Studies by SYRCLE’s tool**

| **STUDY** | **SELECTION BIAS** | | | **PERFORMANCE BIAS** | | **DETECTION BIAS** | | **ATTRITION BIAS** | **REPORTING BIAS** | **OTHER** |
| --- | --- | --- | --- | --- | --- | --- | --- | --- | --- | --- |
|  | **Sequence generation** | **Baseline characteristics** | **Allocation concealment** | **Random housing** | **Blinding** | **Random outcome assessment** | **Blinding** | **Incomplete outcome data** | **Selective outcome reporting** | **Other sources of bias** |
| Tufail et al 2010 | unclear | yes | unclear | unclear | unclear | yes | unclear | yes | unclear | yes |
| Yoo et al 2011a | unclear | yes | unclear | unclear | unclear | yes | unclear | yes | unclear | yes |
| Yoo et al 2011b | unclear | yes | unclear | unclear | unclear | yes | no | yes | unclear | yes |
| Deffieux et al 2013 | unclear | yes | unclear | yes | unclear | yes | unclear | yes | yes | yes |
| Kim et al 2013 | unclear | yes | unclear | unclear | unclear | yes | unclear | yes | yes | yes |
| Kim et al 2014a | unclear | yes | unclear | unclear | unclear | yes | unclear | yes | unclear | yes |
| Kim et al 2014b | unclear | yes | unclear | unclear | unclear | yes | unclear | yes | unclear | yes |
| Kim et al 2015 | unclear | yes | unclear | unclear | unclear | unclear | unclear | yes | unclear | yes |
| Chu et al 2015 | unclear | yes | unclear | unclear | unclear | unclear | unclear | yes | yes | yes |
| Guo et al 2015 | unclear | yes | unclear | unclear | yes | yes | yes | yes | yes | yes |
| Lee et al 2014 | unclear | yes | unclear | unclear | unclear | unclear | unclear | yes | unclear | yes |
| Lee et al 2016 | unclear | yes | unclear | unclear | unclear | unclear | unclear | yes | yes | yes |
| Yu et al 2016 | unclear | yes | unclear | unclear | unclear | unclear | unclear | yes | yes | yes |
| Wattiez et al 2017 | unclear | yes | unclear | yes | unclear | yes | unclear | yes | unclear | yes |
| Dallapiazza et al 2017 | unclear | yes | unclear | unclear | unclear | unclear | unclear | yes | unclear | yes |
| Guo et al 2018 | unclear | yes | unclear | unclear | unclear | yes | unclear | yes | unclear | yes |
| Sato et al 2018 | unclear | yes | unclear | unclear | unclear | yes | yes | yes | yes | yes |
| Yang et al 2018 | unclear | yes | unclear | unclear | unclear | unclear | unclear | yes | yes | yes |
| Yoo et al 2018 | unclear | yes | unclear | unclear | unclear | yes | unclear | yes | unclear | yes |
| Zhang et al 2018 | unclear | yes | unclear | yes | unclear | unclear | yes | yes | yes | yes |
| Li et al 2018 | yes | yes | unclear | unclear | unclear | unclear | unclear | yes | yes | yes |
| Daniels et al 2018 | Unclear | yes | unclear | unclear | unclear | unclear | unclear | yes | yes | Yes |
| Xie et al 2018 | unclear | yes | unclear | unclear | unclear | unclear | unclear | yes | yes | yes |
| Sharabi et al 2019 | unclear | yes | unclear | unclear | unclear | unclear | unclear | yes | yes | yes |

**Table S2. Quality Assessment of Included Human Studies by PEDro**

| **Study** | **Random allocation** | **Concealed allocation** | **Baseline comparability** | **Blind subjects** | **Blind therapists** | **Blind assessors** | **Adequate follow-up** | **Intention-to-treat analysis** | **Between group comparisons** | **Point estimates and variability** | **Total Scores** |
| --- | --- | --- | --- | --- | --- | --- | --- | --- | --- | --- | --- |
| Hameroff et al 2013 | 1 | 0 | 1 | 1 | 1 | 0 | 1 | 1 | 1 | 1 | 8/10 |
| Legon et al 2014 | 1 | 0 | 1 | 1 | 0 | 1 | 1 | 1 | 1 | 1 | 8/10 |
| Lee et al 2015 | 1 | 0 | 1 | 1 | 0 | 1 | 1 | 1 | 1 | 1 | 8/10 |
| Lee et al 2016a | 1 | 0 | 1 | 1 | 0 | 1 | 1 | 1 | 1 | 1 | 8/10 |
| Lee et al 2016b | 1 | 0 | 1 | 1 | 0 | 1 | 1 | 1 | 1 | 1 | 8/10 |
| Ai et al 2016 | 0 | 0 | 0 | 0 | 0 | 0 | 1 | 1 | 0 | 1 | 2/10 |
| Legon et al 2018a | 1 | 0 | 1 | 0 | 0 | 0 | 1 | 1 | 1 | 1 | 6/10 |
| Legon et al 2018b | 1 | 0 | 1 | 1 | 0 | 0 | 1 | 1 | 1 | 1 | 7/10 |
| Ai et al 2018 | 0 | 0 | 1 | 0 | 0 | 0 | 1 | 1 | 1 | 1 | 5/10 |
| Gibson et al 2018 | 1 | 0 | 1 | 1 | 0 | 0 | 1 | 1 | 1 | 1 | 7/10 |

Notes: Monti et al 2016 was a single-case study, hence we did rate this article by PEDro
